# Distribution and Expansion of Alien Fish Species in the Karun River Basin, Iran

**DOI:** 10.3390/fishes8110538

**Published:** 2023-10-31

**Authors:** Mojgan Zare Shahraki, Yazdan Keivany, Eisa Ebrahimi Dorche, Karen Blocksom, Andreas Bruder, Joseph Flotemersch, Doru Bănăduc

**Affiliations:** 1 Department of Natural Resources, Isfahan University of Technology, Isfahan 84156-83111, Iran; 2 U.S. Environmental Protection Agency, Office of Research and Development, Corvallis, OR 97333, USA; 3 Institute of Microbiology, University of Applied Sciences and Arts of Southern Switzerland, via Flora Ruchat Roncati 15, 6850 Mendrisio, Switzerland; 4 U.S. Environmental Protection Agency, Office of Research and Development, Cincinnati, OH 45268, USA; 5 Applied Ecology Research Center, Lucian Blaga University of Sibiu, 550024 Sibiu, Romania

**Keywords:** Karun Basin, fish, human impact, alien species, redundancy analysis, habitat quality

## Abstract

We assessed the distribution of alien fishes in the Karun River Basin, Iran. Fish were collected from 39 sites during the November–December 2018 low-flow period. In total, 39 fish species from nine orders and 14 families were documented. Among these, 10 species were alien to the basin (986 individuals; 15.7%). Four species were the most abundant alien species and primarily in impounded, downstream reaches. Redundancy analysis (RDA) was conducted to identify the extent of changes in alien fish assemblages with environmental parameters. RDA1 and RDA2 accounted for 36.24% and 25.33% of the variation of alien species, respectively. Altitude, depth, electrical conductivity, water temperature, turbidity, dissolved oxygen, and river width were the most significant parameters affecting alien species distributions. We present a dual-pathway cause-and-effect hypothesis proposing that alien fish species presence causes declines in the ecological status of native fish communities. We then explore how human-induced aquatic ecosystem degradation creates opportunities for alien species to invade new ecosystems, further impacting native fish communities. Our study contributes insight into the cause and effect of the presence of alien fish species in the Karun River Basin and emphasizes the urgency of conservation measures to protect this critically endangered watershed.

## 1. Introduction

A large number of stressors induce major threats and risks to aquatic habitats and their living and non-living elements and structures globally [1–5]. Consequently, fishes, as a vital group of organisms in aquatic ecosystems [6–8], are in decline. They suffer secondary losses due to the diverse impacts of human activities [9–22]. Global concern is growing as biodiversity plays a crucial role in ecosystem function and resilience [1,3,23]. Over the last 200 years, alien species have increased their range by 37% and this expansion shows no signs of stopping [24]. Seebens et al. [25] predict a further 36% increase in the number of alien species established globally by 2050. This is concerning as the dynamic equilibrium of an ecosystem can be disrupted by alien species introduction [26,27]. Consequently, alien species are widely considered to be one of the main threats to biodiversity and the second leading cause of animal extinctions [28]. Declines are particularly noteworthy in freshwater ecosystems, primarily due to habitat destruction, pollution, overexploitation, and the introduction of alien species [3,29]. Trends in socio-economic development suggest that the introduction of alien fish will persist, along with the associated environmental risks and biodiversity losses [30].

By definition, “an alien species is any species intentionally or accidentally transported and released by humans outside its native current range” [31]. The introduction of alien freshwater fish species has been practiced since the late 1800s [32,33] to support aquaculture for food and aquarium fish production, stocking of ecosystems to control disease vectors, and to support recreational fishing [34,35]. Once introduced, alien species easily spread to neighboring systems, including those of adjacent countries [35]. The success of alien species in freshwater ecosystems is often attributed to their broader environmental and physiological tolerance [36,37]. However, the specific mechanisms and impacts of alien species differ across ecosystems, species, and spatial scales [30,38–46]. As a result of these impacts on native populations, the presence and relative abundance of alien fish species can function as an indicator of biological integrity [47,48].

In recent decades, several alien fish species have been introduced in Iranian waters [49]. Among these are the blue tilapia, *Oreochromis aureus* (Steindachner 1864), and redbelly Tilapia, *Coptodon zillii* (Gervais 1848), [50] that entered the waters of Iran via transboundary waters due to inadequate precautions to prevent their spread [51–55]. An additional 23 to 32 alien fish species have been documented in Iranian freshwater ecosystems [56–58].

Focusing specifically on the Karun River Basin, a comprehensive analysis of the status of all native and endemic fish species has been previously provided [48]. This paper focuses specifically on the distribution and factors influencing the expansion of alien fish species in the same study area. This is relevant as the Karun River Basin provides crucial ecosystem services (e.g., drinking water, irrigation for agriculture, support for industries and hydropower plants) [58] to those in the basin and beyond. Subsistence fishing, which can be significantly affected by the presence of alien fish species, is of special concern as it is an integral part of rural and urban livelihood systems in the Karun Basin. It is easy to conceive how the presence of alien fish species contribute to the decline in the ecological status of native fish communities, but the human-induced degradation of aquatic ecosystems that enhances opportunities for these aliens to penetrate new ecosystems must also be considered. This dual-way cause-and-effect working hypothesis forms the foundation of this research [54,55]. We examine the relationship between alien fish data and environmental variables at sampling sites. We then discuss the potential negative impacts of invasive alien species on native fish communities of the basin which include many endemic species. This information is essential for informed decision making and effective environmental management of the Karun River.

## 2. Materials and Methods

### 2.1. Study Area

**Figure 1.**
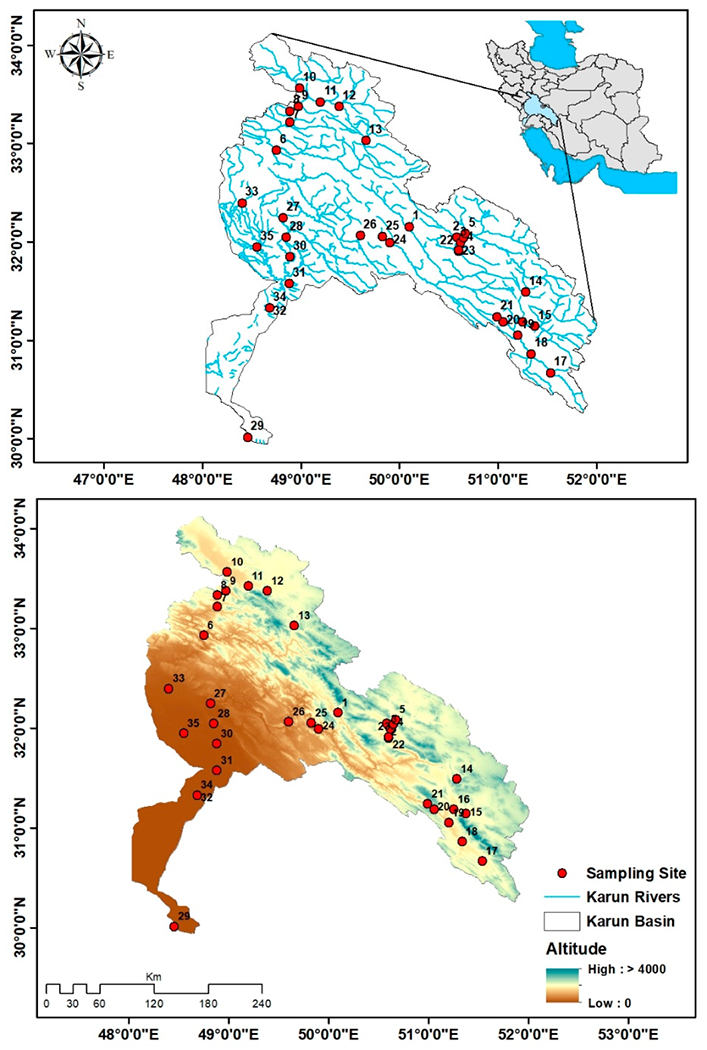
Map of the study area and sampling site locations.

The Karun River Basin is located in southwestern Iran and encompasses seven provinces (Chaharmahal-va-Bakhtiari, Fars, Isfahan, Khuzestan, Kohgiluyeh-va-Boyer-Ahmad, Lorestan, and Markazi) [59] (Figure 1).

It is Iran’s largest river system and covers 67,000 km^2^, i.e., 4.2% of Iran’s area [60,61]. Its main stream is more than 950 km long with an average annual flow of about 575 m^3^/s in Ahvaz city which is located in the downstream extent of the system. The headwaters of the Karun are in the Zagros mountains in the north and flow into the Arvandrud River (Tigris–Euphrates Basin) before discharging into the Persian Gulf. The river system has a slope of 0–8.5% in the low- and high-altitude areas. Water abstraction is used for irrigation of agricultural lands that covers more than 380,000 ha [62]. The altitude ranges from sea level to >4000 m above the sea level (m.a.s.l.), which, along with its topography, creates diverse climatic conditions. According to the Koppen–Geiger climate classification, the Karun River has four different sub-type climatic conditions including arid, semi-arid, Mediterranean, and humid continental climatic conditions [63]. The catchment has an average annual rainfall of 600 mm and an air temperature that ranges from 20 to more than 50 °C in summer and from <0 to 30 °C in winter [64].

### 2.2. Fish Sampling

Fish were collected in the Karun River Basin at 35 sites (Figure 1) during the low-flow period in November and December 2018. Sites 1 to 26 were positioned in wadable river sections and were sampled using backpack electrofishing equipment (model: Samus 1000) and a dip net. Sites 27 to 35 (non-wadable sites) were positioned in downstream basin areas and sampled using a boat electrofishing system and a dip net (hand-made for electrofishing with a power input of 220 V and output power of 100–500 W). At each site, the reach sampled was approximately 200 m in length and included available mesohabitats (e.g., riffles, runs, and pools). We standardized the catch per unit effort (CPUE) on distance rather than time because the effort (i.e., time) required to safely navigate the stream habitats varied greatly among sites, prohibiting the use of time as a standardizing factor. We used species detection curves to affirm the adequacy of sampling (Appendix A) [65]. The fish sampling effort at each site was approximately 90 ± 20 min [48,64]. Although this standardization does not prevent methodological differences in fishing efficiency at different sites with consequences on the dataset, we chose this procedure as the best compromise. Additionally, we retained a certain number of individuals per species in formaldehyde 10% and transferred them to the laboratory at Isfahan University of Technology for further examination and verification of the field identification. Subsequently, the remaining fish were released back into the stream at the original capture site. With the assistance of locally relevant identification guides, and expert ichthyologists, we successfully identified all fish species based on morphological characteristics [49,66–68]. At each site, the biological data collected included the occurrence (presence or absence) and abundance of alien, native (species that evolved in the Karun and other rivers), and endemic fish species (species that are restricted in distribution to the Karun Basin).

### 2.3. Water Sampling

We collected triplicate surface water samples at 10–15 cm depths at all the 35 sites with pre-washed (HCl 2%) plastic containers. Samples were transported to the laboratory at Isfahan University of Technology (IUT) for water chemistry analysis. A portable multiparameter probe (Oxi, 3205, WTWWeilheim, Germany) was used for in situ measurements of dissolved oxygen concentrations and water temperature. Other variables such as pH, electrical conductivity (μmho/cm), hardness (mg/L CaCo_3_), total suspended solids (mg/L), total dissolved solids (mg/L), nitrate (mg/L), nitrite (mg/L), phosphate (mg/L), alkalinity (mg/L CaCo_3_), biological oxygen demand (mg/L), and chemical oxygen demand (mg O_2_/L) were analyzed in the lab using standard methods. Data were also collected on general habitat features such as river width and depth. Altitude was also recorded at all sampling sites from GPS information (Garmin GPSMAP 64X).

### 2.4. Data Analysis

Detrended Canonical Correspondence Analysis (DCCA) was run to identify the most relevant response model (between linear or unimodal) for gradient analysis [47,69–71]. A linear model, redundancy analysis (RDA), was selected rather than a unimodal method (e.g., Canonical Correspondence Analysis—CCA) because the dominant gradient length was less than 3 [70,72]. Redundancy analysis (RDA) was performed as a direct gradient method to define the amount of variation in alien fish communities that could be described by environmental parameters [73,74]. Based on the “forward selection method” and ordiR2step function, the most important variables were selected based on significance and adjusted squared R. Of the ten alien fish species collected, only five species (with a frequency of occurrence higher than 5% [72]) and 12 environmental variables were selected (after a forward stepwise selection) for inclusion in the RDA.

To determine the optimal and less favorable ecological conditions in the Karun River, we utilized the Least-Disturbed method, which characterizes the “best-available physical, chemical, and biological habitat conditions given the current state of the waterbody”. Essentially, the least-disturbed sites represent areas where biota experience minimal exposure to the prevailing stressor gradients [75]. Therefore, we performed Principal Component Analysis (PCA) on 16 standardized and centered variables including physicochemical and habitat characteristics. PCA is a valuable tool for identifying factors and sources that may affect water systems and cause changes in water quality. The first axis (PC1) derived from the PCA was identified as the primary stressor gradient, following the approach by Blocksom and Johnson [75]. By observing the stressor’s direction along PC1, sites falling within the 25th quartile of the PC1 were categorized as least disturbed, those within the 75th quartile as most disturbed, and sites between the two quartiles as moderately disturbed sites [65,76]. Afterwards, we generated box plots to compare the biomass and abundance values of native, endemic, and alien fish species across the least, moderate, and most disturbed sites of the Karun Basin. To examine significant differences, we conducted an analysis of variance (ANOVA) using the Tukey HSD method. All statistical analyses were conducted utilizing Microsoft Excel 2016 and R software (v. 4.0.4) [77]. The vegan (2.5–6) [78] and ggplot2 (v. 2.2.0) [79] packages were used for analysis and graphics. The study area map was generated using ArcGIS 10.2 software [80–82].

## 3. Results

### 3.1. Alien Fish Species Composition

**Figure 2.**
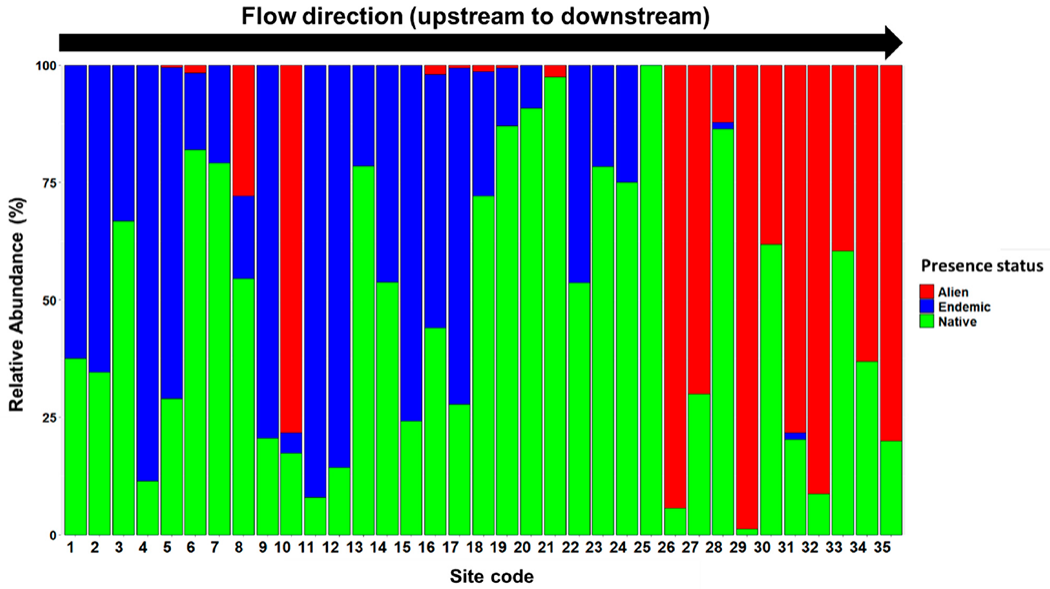
The relative abundance of individuals (%) of endemic, native, and alien species at different sampling sites in the Karun River Basin, Iran.

**Figure 3.**
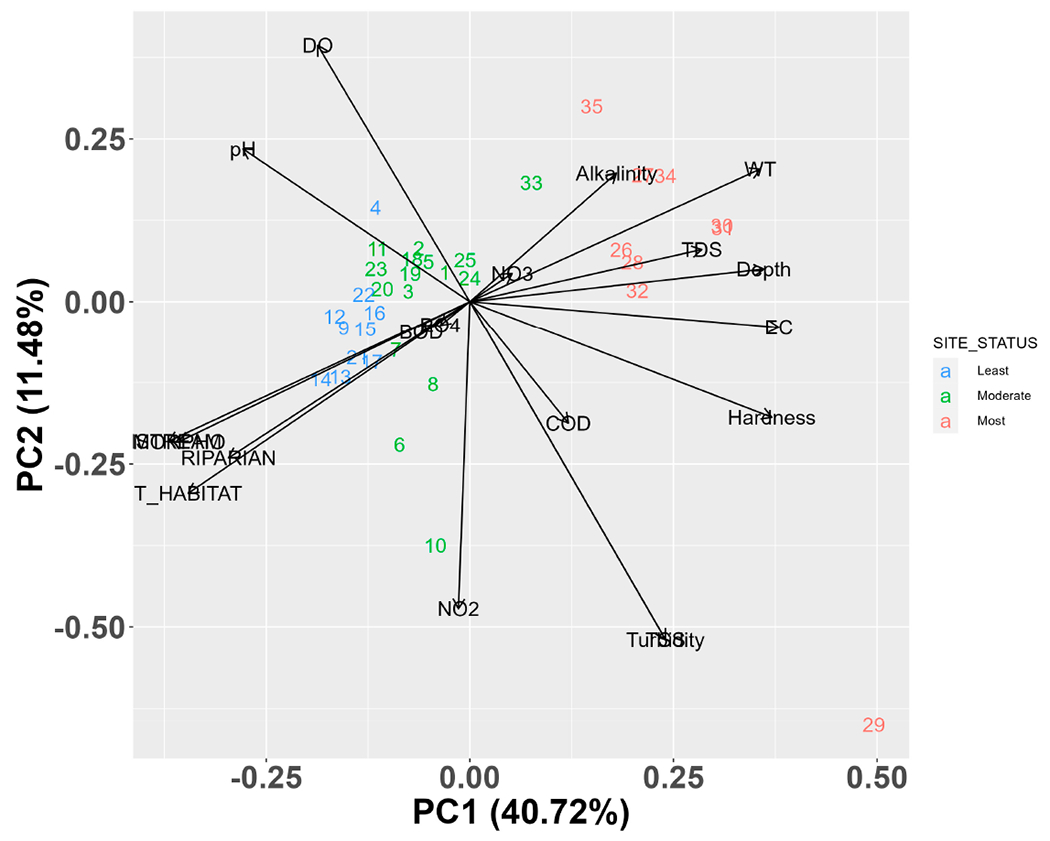
Principal Component Analysis (PCA) plot in the Karun River Basin, Iran. (Abbreviation: DO: dissolved oxygen; BOD: biological oxygen demand; COD: chemical oxygen demand; NO_3_: nitrate; TDS: total dissolved solids; WT: water temperature; EC: electrical conductivity; NO_2_: nitrite, instream, morphological, and total habitat scores.)

**Figure 4.**
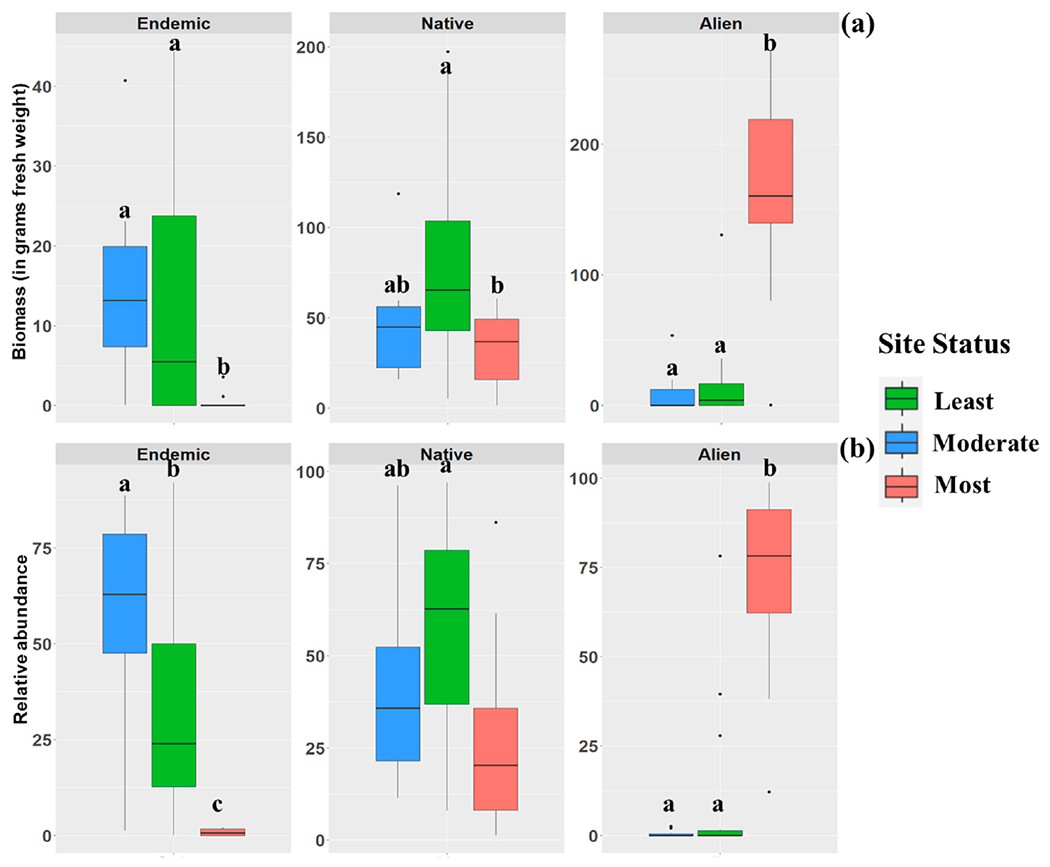
(**a**) The biomass (in grams of fresh weight) and (**b**) relative abundance of alien, native, and endemic fish species at the least, moderate, and most disturbed sites of Karun River Basin, Iran. Letters above boxplots show statistically different groups.

A total of 6272 fish representing nine orders, 14 families, 30 genera, and 39 species of bony fishes were collected (Table 1). Of these, 29 were native species (12 endemic to the basin) and 10 were alien species (Table 1). Appendix B provides a comprehensive record of all observed fish species, indicating their presence or absence. Among these, 3387 (54%) were native, 1899 (30.3%) were endemic, and 986 (15.7%) were alien fish species. Proportions of these groups varied among sampling sites (Figure 2). The PCA results revealed that the majority of the downstream sites were categorized as the most disturbed locations (Figure 3). As a result, the biomass and abundance of alien fish species were significantly higher in the most disturbed sites in comparison to native and endemic fish species (Figure 4). Alien fish species occurred at 19 of 35 sites, particularly in the downstream sites, whereas they were absent from most headwaters (Figures 2 and 4). Among the alien species, *Gambusia holbrooki* and *Pseudrasbora parva* were found only at site 10 (i.e., Tireh), while *Carassius gibelio* was present in the upper and lower sections of the Karun River Basin. Some alien species such as *Oreochromis aureus, Hemiculter leucisculus*, and *Coptodon zillii* were present only in downstream areas of the system. Appendix C provides photos of all observed alien fish species in the Karun River Basin.

### 3.2. Relationships between Alien Fish Assemblages and Environmental Variables

**Figure 5.**
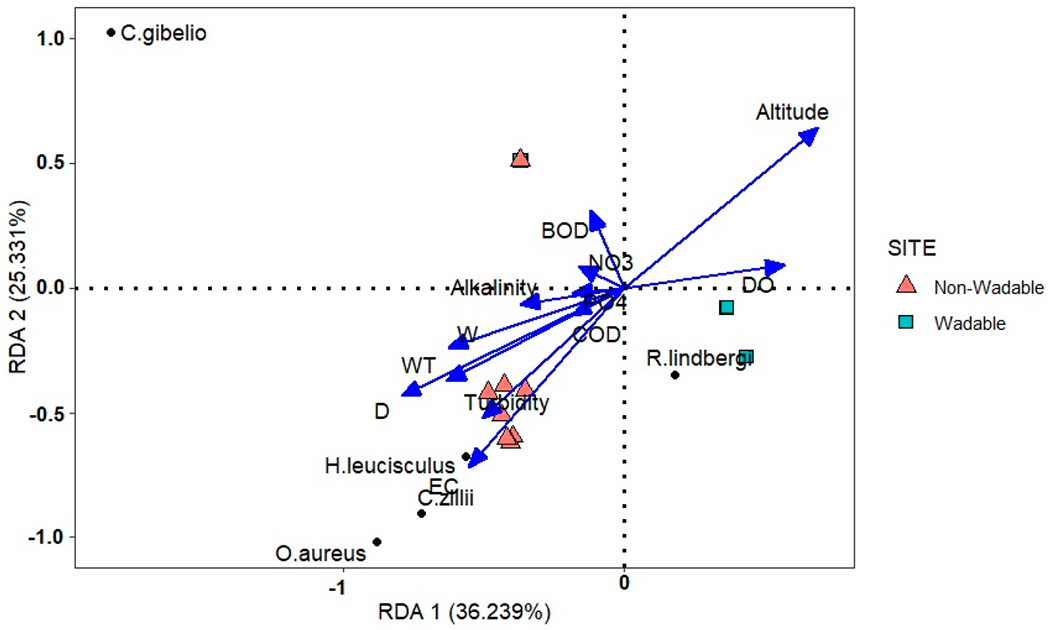
Redundancy analysis ordination between alien fish assemblages and environmental variables in the Karun River Basin, Iran (abbreviations are explained in Table 3).

The average values of the environmental variables measured in the Karun River Basin are presented in Table 2. The first two axes (RDA1 and RDA2) accounted for 36.24% and 25.33% of the variation for five alien fish species, respectively (Figure 5). Altitude, depth (D), electrical conductivity (EC), water temperature (WT), turbidity, dissolved oxygen (DO), and width (W) were the most influential and significant variables affecting the distribution of alien fish species in the study area (Table 3). Different fish species preferred different environmental conditions. For example, the presence of *O. aureus, C. zillii*, and *H. leucisculus* was positively correlated with EC, turbidity, water temperature, width, and depth of the river, but it was negatively correlated with altitude. The dominate substrate at sites varied from mud to small boulders (Appendix D).

## 4. Discussion

Recent decreases in water quality in the downstream sections of the Karun River Basin due to reduced water flow and pollution by urban sewage, in conjunction with spawning habitat degradation, have resulted in the decreased survival of native and endemic fish species such as *Luciobarbus esocinus, Luciobarbus barbulus, Barbus lacerta*, and *Barbus karunensis* [83]. Current research documented the presence of ten alien species from seven families in the Karun River Basin. Most of these species are considered to be relatively tolerant to river impairment, which also makes them useful candidates as indicators of the declining river health [47,84].

**Figure 6.**
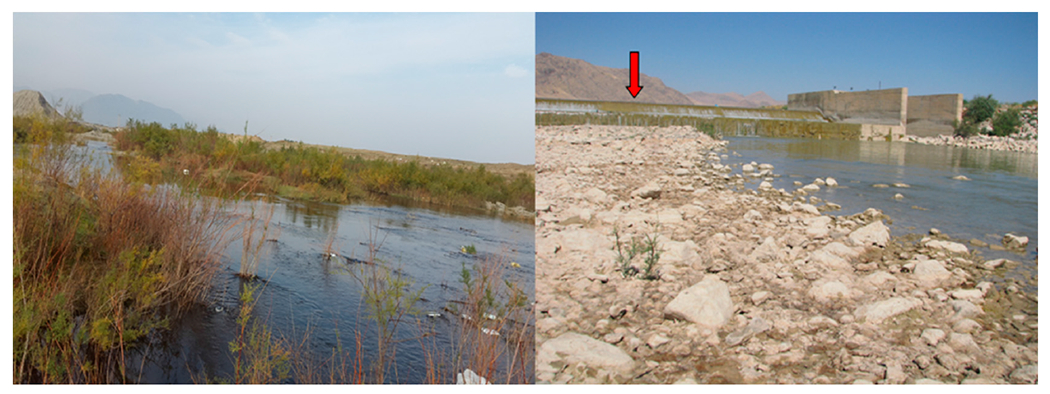
The presence of small ponds and physical barriers at site 10 in the Karun River Basin (red symbol shows the barrier at this sampling site).

Water temperature was among the environmental factors revealed as contributing to the distribution of alien species in the Karun River. For instance, some alien species (e.g., *O. aureus* and *C. zillii*) may not be as competitive at cooler temperatures, which may explain why their presence was limited to the downstream parts of the catchment having higher temperatures [85–87]. The distributions of *O. aureus, C. zillii*, and *H. leucisculus* populations were correlated with high turbidity and decreasing water transparency. This finding corroborates those of other studies showing the presence of alien species in waters with poor quality [42,86–88]. Site 10 (Tireh) in the upstream section of the study area (sites 1–25) had substantially more alien species than other upstream sites. At this sampling site, it is believed that the presence of certain alien species (e.g., *R. lindbergi, G. holbrooki*, and *P. parva*) can be attributed to organizations introducing commercial carp into dams or wetlands as part of their ranching program. Small ponds of different sizes with obstacles limiting access to the main river, and the existence of physical barriers in the main channel of the river, have been put in place to limit further distribution of these species (Figure 6). Taylor et al. [89–91] suggested habitat alteration to help control the spread of non-native species in North America.

Four species (*O. aureus, C. zillii, H. leucisculus*, and *C. carpio*) were only found in lower altitudes in urbanized areas. In these downstream sections, there are many hydroelectronic power plants with dams that control water level and alter flow velocity and habitat types which modify the spatial structure of the fish community structure in an indirect way [92]. The expansion of these species into shallower upstream sections of the basin has likely not occurred for two reasons. First, these species prefer slow flow velocities and warmer temperatures like those that prevail in the impounded deep-water habitats of the downstream parts of the basin [67]. Second, reduced hydrological connectivity due to dams and other structures likely impedes the spread of alien species in the basin, as suggested by only sporadic occurrence in upstream reaches (e.g., site 10); although in this case, they were probably actively introduced [93]. If anthropogenic impacts creating these habitat conditions (e.g., climate change, water abstraction) extend further upstream, these species will likely extend their occupancy to those areas. Additional human disturbances (e.g., contamination, river modifications, and flow regulation) could likewise contribute to upstream expansion as they can lead to higher conductivity, muddy substrates, lower riparian vegetation cover, lower dissolved oxygen concentrations, and the presence of aquatic macrophytes and filamentous algae [47,53,82,94].

### Potential Origin, Possible Destructive Effects, and Management of Alien Fish Species Observed in the Karun River Basin

In the downstream sections (sites 27 to 35) of the Karun Basin, the high relative abundance and biomass of alien species have contributed to decreases in the relative abundance of native species, including many endemics (Figures 4 and 5). In recent years, tilapia (*C. zillii* and *O. aureus*), which are native to Africa, entered Iranian waters from Iraq and/or through accidental or intentional introductions [51]. Tilapia are omnivorous fish that feed at lower trophic levels, which makes them much less expensive to feed and breed than other fish species and explains their widespread use in aquaculture [95]. In the Khuzestan Province of Iran, as well as in other countries, tilapia have also been actively introduced to control aquatic plants in sugarcane effluent drainage channels [96,97]. The species, once escaped from their point of initial introduction, might affect native aquatic plants and cause structural impacts on entire ecosystems [97]. According to anecdotal information from local fishermen, tilapia now dominate the catch in downstream sections of the Karun Basin. Their adaptation to high temperature, low dissolved oxygen, and salinity fluctuations [46,51,98,99], combined with reproduction strategies which include paternal care, rapid growth rate, high fertility, and omnivorous feeding habits, allows tilapia to efficiently colonize areas outside their native range [72,100–103]. Due to their competitive advantages, tilapia can then outcompete native and endemic species and cause fish community structure changes as shown in this study and other studies [47,104]. Sing et al. [46] reported that Nile tilapia (*Oreochromis niloticus*) reduced the catch of native carp species in the Ghana River while increasing overall fish production in the system. Due to the negative impact upon native and endemic species [46,105], the status and range of these invasive species should be monitored in Iranian waters.

The spread of tilapia can be controlled using biological methods, i.e., by supporting other organisms, ideally endemic and other native fish species. For example, predatory fishes such as the native *Silurus triostegus* and *Leuciscus vorax* have contributed to reductions in tilapia populations (especially juvenile tilapia) in the Khuzestan Province [97,106,107]. Management practices supporting their presence in the system would help address the issue. This is important because fishing nets used to catch adult tilapia are not effective for capturing juvenile tilapia [97]. Without such management measures, it is likely that tilapia populations would further increase and expand into the upstream sections of the Karun River Basin.

Prussian carp (*Carassius gibelio*), originally from Siberia, is an alien species that was caught at different sampling sites throughout the Karun Basin. It is reported from rivers and ponds in neighboring countries like Iraq [52] but also in Europe [89,108]. Based on its biological features and requirements, it outcompetes many cyprinid species for food and habitat. This can reduce population sizes and promote the local extinction of some native/endemic fish species [34,109]. Prussian carp have unique reproductive traits partly explaining their competitive advantage. They have eggs that can be induced by sperms of other cyprinid species, allowing the production of offspring in the absence of conspecific males [34,108,110,111]. Given that a substantial trade of this species as a decorative fish is ongoing, this species is still being traded in Iran, so managers and authorities in Iran should be mindful of its potential environmental impacts [34]. For instance, Azevedo-Santos et al. [112] highlighted a potentially effective method to prevent fish introductions in Brazil: promoting educational opportunities that foster a change in human behavior.

The native range of Sharpbelly (*Hemiculter leucisculus*) extends from southern Russia to southern Korea via China and Vietnam [113], and it is likely to have been accidentally introduced to Iran from Central Asia with commercial shipping. This species was detected only in downstream sections of the Karun Basin and may be related to higher water temperature and lower water velocity. It is noted for having a strong dorsal fin spine which serves as a deterrent to predators. It may compete with native species and may also feed on their eggs and fry [36]. Due to its greater resistance to predation, high fertility, and omnivorous feeding preferences, it has replaced native fish in the Aral Sea Basin [114] and likely presents a risk to native and endemic species in the Karun River Basin.

The common carp (*Cyprinus carpio*), native to Eurasia, is a farmed fish and is widely found in all freshwater resources of Iran [115]. In our study, its occurrence was fewer than five individuals at one site and we did not consider it in our data analyses. However, according to the local fishermen, it is abundant in their catches from this basin. This discrepancy is likely due to common carp being more susceptible to the fishing nets used by local fisherman than the electrofishing approaches used in this study [116–118]. The species is an omnivorous bottom feeder in rivers and lakes and its presence is noted for resulting in increased siltation, decreased water quality, and influences on native flora and fauna [119]. It is associated with the decline and local disappearance of native and endemic species in Argentina, Australia, Venezuela, Mexico, Kenya, India, etc. [120], and it should be monitored in the Karun River Basin.

The Amur goby (*Rhinogobius lindbergi*) was described from the Amur and Ussuri rivers, Russia [121]. It was probably accidentally introduced to Iranian inland waters along with non-native Cyprinids for the Iranian aquaculture industry [122]. In the upstream part of sites 26 and 27 are the Masjed Soleiman and Gotvand hydroelectric power plant, which are opened and closed daily. One could argue that the lack of stable water flow and substrate has produced environmental conditions undesirable for most fish species yet adequate for the presence of this species.

The eastern mosquitofish (*Gambusia holbrooki*), which is native to the United States [123], has been introduced to Iran, Iraq, Türkiye, and Syria to control larval mosquitoes and reduce malaria outbreaks [52,124]. In our study, it was found only in one site (site 10) with shallow and slow water. Individuals of this species are fertile breeders, are able to enter into the microhabitats of rare and native species, and are often reported as predators [125,126]. Eastern mosquitofish feed on eggs of fish including those of economically valuable species but also those of endangered native species, amphibians, and invertebrates [126]. Taybi et al. [126] further reported that in disturbed areas, *G. holbrooki* is often abundant because of a wide tolerance to unfavorable abiotic conditions. Considering the negative impacts (aggressive and predatory behavior) of this species, the famous ichthyologist Myers (1965) called it the “fish destroyer”.

The stone moroko (*Pseudorasbora parva*), which is an East Asian cyprinid species, was found in only one shallow site (site 10, Figure 6) with moderate water quality (Figure 3). Ekmekçï and Kirankaya [125] described it as an opportunistic species with great ecological and physiological tolerance, also tolerant to moderate contamination, high temperature, and low water levels. An important factor contributing to the rapid distribution of this species can be related to the spawning ability on different soft substrates and competition for food with native and endemic species [36,125]. Furthermore, it is considered a serious threat due to risk for disease transmission and reproduction inhibition of *Leucaspius delineates* (endangered species) in Europe [30,43]

Grass carp (*Ctenopharyngodon idella*), a species native to East Asia [108], were found at a sampling site covered by macrophytes in the littoral zone (site 28). It can damage the spawning substrate of phytophilous fish species by feeding on macrophytes and thereby affecting some native and endemic species in the Karun River Basin such as *Capoeta aculeata, Squalius berak, Squalius Lepidus*, and *Alburnoides idignensis* [127–129].

Rainbow trout (*Oncorhynchus mykiss*) were introduced as a relevant species for aquaculture and recreational angling, and they are noted as being one of the main predators of eggs and small individuals of native species [130,131]. The species was present at Kata (site 16) which is close to a rainbow trout aquaculture facility. *Oncorhynchus mykiss*, a salmonid native to the North American west coast, is one of the first species considered to be almost globally invasive. It currently exists in more than 90 countries [108,132]. It is a very common alien species in Iranian freshwaters which it colonized after escaping from aquaculture facilities [131].

## 5. Conclusions

The main goal of the current study was to update the status of alien fish species distribution in the Karun River Basin in support of improved biodiversity conservation. Invasive species are a major biodiversity threat due to their extensive tolerance to unfavorable conditions and ability to replace native species. Ecological and biological threats are mainly caused by invasive species that are either generalists or sufficiently adapted to the prevailing natural conditions of non-native ecosystems and show high reproductive rates. Under the appropriate situations, some alien species such as *C. gibelio*, *O. aureus*, and *C. zillii* produce large populations and exert significant pressure on populations of native and endemic fish species in the Karun River Basin, including *Carasobarbus kosswigi, Arabibarbus grypus*, *Barbus karunensis*, *Capoeta coadi*, and *Luciobarbus barbulus*.

The drastic decline in important native fish species, concomitant with the rapid invasion and establishment of alien fish, most notably in downstream sections of the Karun Basin, is receiving increased attention from scientists, conservation entities, and the Iranian government. The collection of scientifically robust data on the occurrence and extent of alien species in the basin is an important indicator for understanding the drivers of impairment and is critical to monitoring efforts intended to support the protection and recovery of populations of native and endemic species. Habitat restoration activities (e.g., flow modification, woody debris introductions, or bank vegetation restoration) could further improve the ecological conditions required for native fish reproduction and may reduce the competitive advantage of alien fish populations. An increased understanding of the temporal and spatial changes in the fish community structure, and the effects of human and ecological processes that drive these changes, is essential for the development of management policies that will support the protection and recovery of native and endemic fish biodiversity in the downstream sections of the Karun River Basin.

In conclusion, this research has documented the prevalence of alien species in the lower sections of the Karun River which have unquestionably contributed to the declines in the ecological status of local and regional native fish associations and communities. Additionally, it highlights that human activities leading to the deterioration of aquatic ecosystems have a direct impact on native fish associations and communities, thereby facilitating the intrusion of alien species into new ecosystems.

To protect what remains of the native and endemic fishes of the Karun River Basin, urgent steps must be taken to mitigate the degradation of existing conditions and implement preventive measures to prevent the unintentional introduction of additional alien fish species. Some introductions of alien fish species in Iran can be attributed to a lack of awareness among the general public and individuals in the fishery and aquaculture sectors regarding its associated risks. To prevent such further introductions, we suggest the development of educational and promotional programs designed to inform relevant stakeholders and enhance public awareness about the risk posed by introduced species. Furthermore, future research should focus on understanding the specific mechanisms leading to declines in native fish species (e.g., competitive advantage in accessing food resources and habitats, habitat loss) to inform of possible management strategies that may aid in their recovery and mitigate future losses.

## Figures and Tables

**Table 1.** Abundance (N) of all recorded fish species in the Karun River Basin, Iran, and IUCN red list status.

Family	Species	IUCN Status	Status	Relative Abundance
Xenocyprididae	*Hemiculter leucisculus* (Basilewsky, 1855)	Least Concern	Alien	2.18
	*Ctenopharyngodon idella* (Valenciennes, 1844)	Least Concern	Alien	0.02
Danionidae	*Barilius mesopotamicus* (Berg, 1932)	Least Concern	Native	0.57
Gobionidae	*Pseudorasbora parva* (Temminck & Schlegel, 1846)	Least Concern	Alien	0.05
Cyprinidae	*Capoeta coadi* (Alwan, Zareian & Esmaeili, 2016)	Not Evaluated	Endemic	13.78
	*Capoeta aculeata* (Valenciennes, 1844)	Not Evaluated	Endemic	7.32
	*Capoeta trutta* (Heckel, 1843)	Least Concern	Native	6.79
	*Carassius gibelio* (Bloch, 1782)	Not Evaluated	Alien	5.29
	*Arabibarbus grypus* (Heckel, 1843)	Vulnerable/Decreasing	Native	0.14
	*Cyprinus carpio* (Linnaeus, 1758)	Vulnerable	Alien	0.08
	*Carasobarbus luteus* (Heckel, 1843)	Least Concern	Native	0.14
	*Barbus lacerta* (Heckel, 1843)	Least Concern	Native	0.26
	*Barbus karunensis* (Khaefi, Esmaeili, Geiger & Eagderi, 2017)	Not Evaluated	Endemic	0.24
	*Cyprinion macrostomus* (Heckel, 1843)	Least Concern	Native	3.84
	*Luciobarbus barbulus* (Heckel, 1847)	Not Evaluated	Native	0.29
	*Carasobarbus kosswigi* (Ladiges, 1960)	Vulnerable/Decreasing	Native	0.08
	*Garra rufa* (Heckel, 1843)	Least Concern	Native	1.16
	*Garra gymnothorax* (Berg, 1949)	Not Evaluated	Endemic	0.8
Leuciscidae	*Alburnus caeruleus* (Heckel, 1843)	Least Concern	Native	0.13
	*Alburnus sellal* (Heckel, 1843)	Least Concern	Native	19.66
	*Alburnus doriae* (De Filippi, 1865)	Not Evaluated	Endemic	2.65
	*Alburnoides idignensis* (Bogutskaya & Coad, 2009)	Not Evaluated	Endemic	2.10
	*Chondrostoma regium* (Heckel, 1843)	Least Concern	Native	13.95
	*Squalius berak* (Heckel, 1843)	Least Concern	Native	0.92
	*Squalius lepidus* (Heckel, 1843)	Least Concern	Native	0.51
	*Acanthobrama marmid* (Heckel, 1843)	Least Concern	Native	4.94
Nemacheilidae	*Turcinoemacheilus saadii* (Esmaeili, Sayyadzadeh, Özulug, Geiger & Freyhof, 2014)	Not Evaluated	Endemic	0.51
	*Turcinoemacheilus hafezi* (Golzarianpour, Abdoli, Patimar & Freyhof, 2013)	Not Evaluated	Endemic	0.05
	*Oxynoemacheilus euphraticus* (Bănărescu & Nalbant, 1964)	Not Evaluated	Endemic	0.37
Cichlidae	*Oreochromis aureus* (Steindachner, 1864)	Not Evaluated	Alien	4.02
	*Coptodon zillii* (Gervais, 1848)	Least Concern	Alien	2.98
Sisoridae	*Glyptothorax galaxias* (Mousavi-Sabet & Eagderi & Vatandoust & Freyhof, 2021)	Not Evaluated	Endemic	0.62
	*Glyptothorax alidaeii* (Mousavi-Sabet & Eagderi & Vatandoust & Freyhof, 2021)	Not Evaluated	Endemic	0.62
Aphanidae	*Esmaeilius vladykovi* (Coad, 1988)	Not Evaluated	Endemic	1.24
Poeciliidae	*Gambusia holbrooki* (Girard, 1859)	Least Concern	Alien	0.14
Mugilidae	*Planiliza abu* (Heckel, 1843)	Least Concern	Native	0.54
Salmonidae	*Oncorhynchus mykiss* (Walbaum, 1792)	Not Evaluated	Alien	0.03
Gobiidae	*Rhinogobius lindbergi* (Berg, 1933)	Not Evaluated	Alien	0.92
Mastacembelidae	*Mastacembelus mastacembelus* (Banks & Solander, 1794)	Least Concern	Native	0.05

**Table 2.** The results of environmental and physico-chemical variables (Mean ± SD) in the Karun River Basin, Iran.

Variable	Unit	Mean ± SD	Range (Min–Max)
Altitude	Meter above sea level	1061 ± 681	1–1961
Depth (D)	Cm	58 ± 26	25–120
Water temperature (WT)	°C	13.5 ± 3.2	7.2–19.6
Electrical conductivity (EC)	(μmho/cm)	740.7 ± 541.3	259–2186
Turbidity	(mg/L)	68 ± 187	16.8–1149
Width (W)	M	52 ± 48	5–170
Dissolved Oxygen (DO)	(mg/L)	8.4 ± 1.3	5.3–12.6
Alkalinity	(mg/L CaCO_3_)	220 ± 13	201–274
Biological Oxygen Demand (BOD)	(mg/L)	2.19 ± 0.99	0.56–4.7
Nitrate (NO_3_)	(mg/L)	8.55 ± 5.4	3.7–37
Chemical Oxygen demand (COD)	(mgO_2_/L)	14 ± 9.4	0.02–41.7
Phosphate (PO_4_)	(mg/L)	0.52 ± 0.33	0.1–1.89

**Table 3.** Results of the redundancy analysis for the occurrence of alien fish species and environmental variables in the Karun River Basin, Iran. Bold variables are influential in the distribution of alien fish species.

Variable	Axis1	Axis 2	*F*-Ratio	*p*-Value
Altitude	0.70	0.64	12.77	0.005 **
Depth (D)	−0.80	−0.43	12.56	0.005 **
Electrical conductivity (EC)	−0.55	−0.72	10.79	0.005 **
Water temperature (WT)	−0.63	−0.37	7.52	0.005 **
Turbidity	−0.50	−0.52	6.74	0.005 **
Width (W)	−0.63	−0.24	6.32	0.005 **
Dissolved Oxygen (DO)	0.57	0.09	4.50	0.02 *
Alkalinity	−0.37	−0.06	1.78	0.165
Biological Oxygen Demand (BOD)	−0.11	0.30	1.21	0.295
Nitrate (NO_3_)	−0.16	0.08	0.74	0.505
Chemical Oxygen Demand (COD)	−0.18	−0.10	0.56	0.69
Phosphate (PO_4_)	−0.18	−0.02	0.41	0.735
Cumulative percentage of the variance of the species abundance	36.24	25.33		
Cumulative percentage of the relation of species abundance and environmental variables	53.14	37.14		

Note:

** = significant at α = 0.01;

* significant at α = 0.05. Bold rows indicate the most influential variables on distribution of alien fish species.

**Figure A1.**
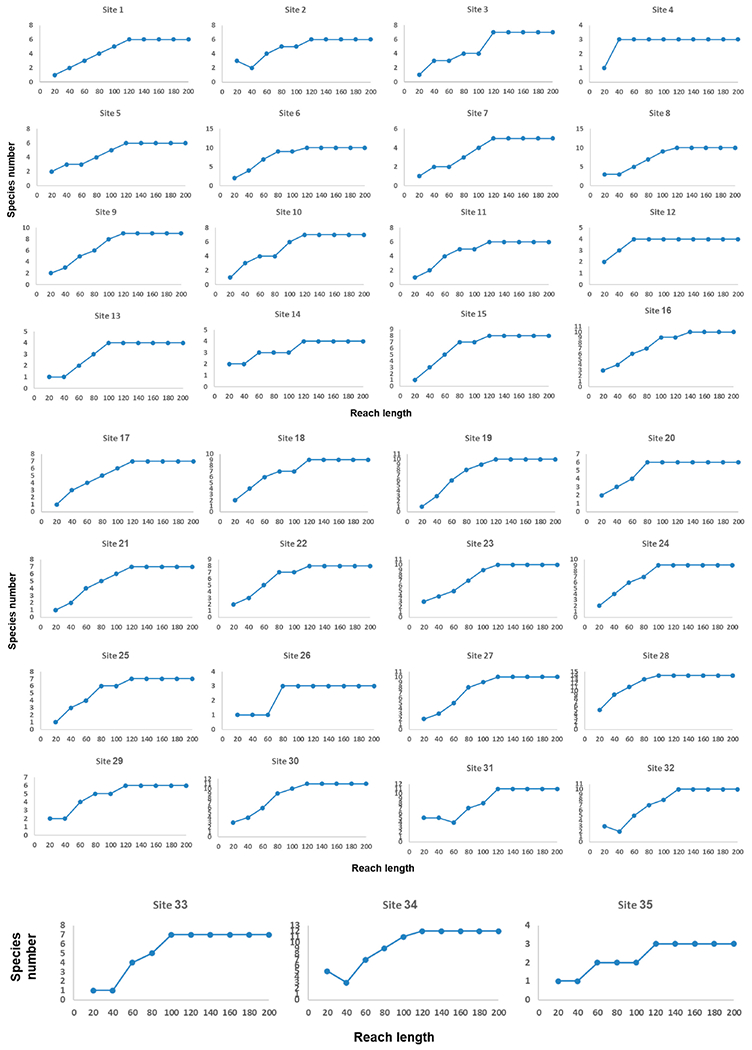
The curve of the number of species in the sampling reach over 90 minutes.

**Figure A2.**
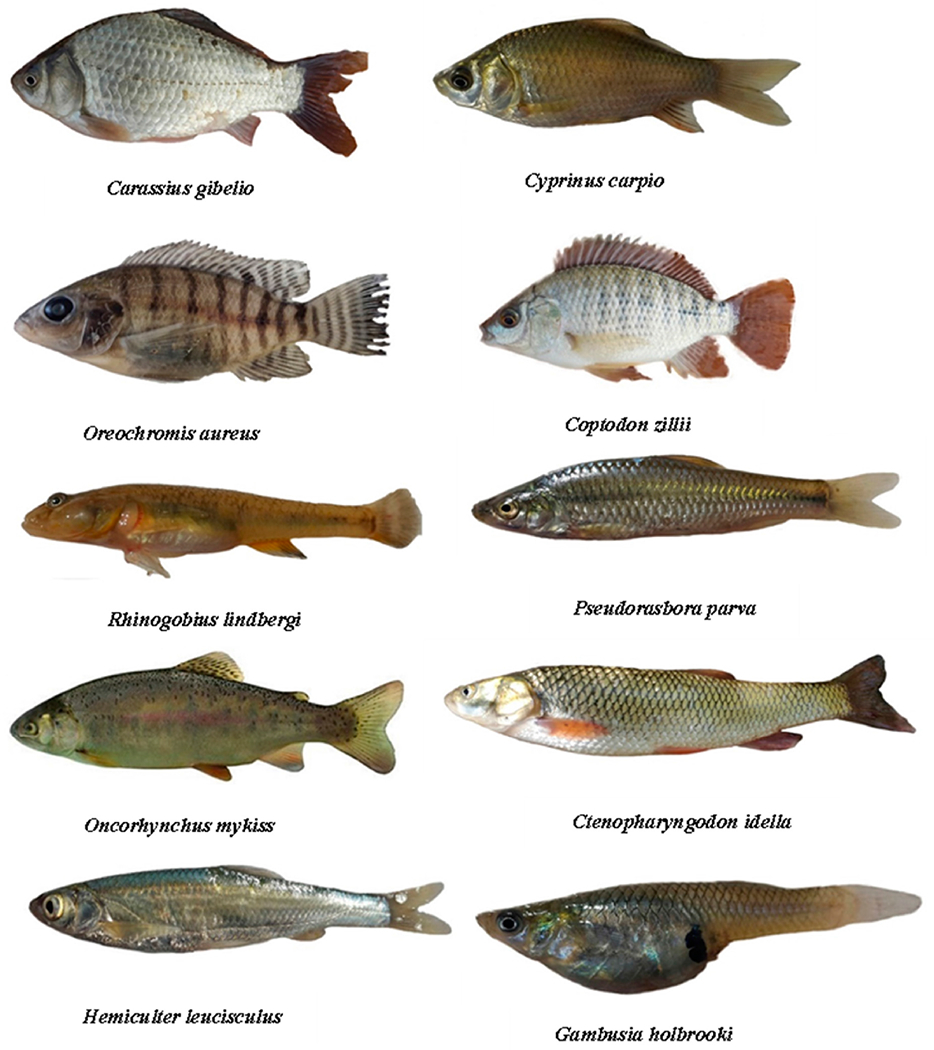
Pictures of Alien Fish Species in the Karun River Basin, Iran. Photo Credit: M. Zare Shahraki.

## Data Availability

There are no supplementary data parts and no publicly archived datasets were analyzed or generated during the study. The data and the location of sampling sites are available on request after publishing.

## Appendix B

**Table A1.** Presence and absence of recorded fish species in the Karun River Basin, Iran.

Family							Cyprinidae										Leuciscidae						Xenocyprididae		Nemacheilidae			Sisoridae		Mugilidae	Aphanidae	Mastacembelidae	Salmonidae	Gobiidae	Gobionidae	Poeciliidae	Cichlidae		Danionidae
Site	*Capoeta coadi*	*Capoeta aculeata*	*Capoeta trutta*	*Carassius gibelio*	*Arabibarbus grypus*	*Cyprinus carpio*	*Garra rufa*	*Garra gymnothorax*	*Barbus lacerta*	*Barbus karunensis*	*Luciobarbus barbulus*	*Carasobarbus luteus*	*Carasobarbus kosswigi*	*Cyprion macrostomus*	*Chondrostoma regium*	*Squalius berak*	*Squalius lepidus*	*Acanthobrama marmid*	*Alburnus sellal*	*Alburnus caeruleus*	*Alburnus doriae*	*Alburnoides idignesis*	*Hemiculter leucisculus*	*Ctenopharyngodon idella*	*Turcinoemacheilus saadii*	*Turcinoemacheilus hafezi*	*Oxynoemacheilus euphraticus*	*Glyptothorax galaxias*	*Glyptothorax alidaeii*	*PlaniPlaniliza abu*	*Esmaeilius vladykovi*	*Mastacembelus mastacembelus*	*Oncorhynchus mykiss*	*Rhinogobius lindbergi*	*Pseudorasbora parva*	*Gambusia holbrooki*	*Oreochromis aureus*	*Coptodon zillii*	*Bariliusmesopotamicus*
1	+	+	+	−	−	−	−	−	−	−	−	−	−	−	+	+	+	−	+	−	−	−	−	−	−	−	−	−	−	−	−	−	−	−	−	−	−	−	−
2	+	+	−	−	−	−	−	−	+	−	−	−	−	−	+	−	−	−	−	−	−	−	−	−	+	−	−	+	+	−	−	−	−	−	−	−	−	−	−
3	+	−	−	−	−	−	+	+	+	−	−	−	−	−	+	−	−	−	−	−	+	−	−	−	+	−	−	−	−	−	+	−	−	−	−	−	−	−	−
4	+	−	−	−	−	−	−	−	−	−	−	−	−	−	+	−	−	−	−	−	+	−	−	−	−	−	−	−	−	−	−	−	−	−	−	−	−	−	−
5	+	+	−	+	−	−	−	−	−	−	−	−	−	−	+	−	−	−	−	−	+	−	−	−	−	−	−	−	−	−	+	−	−	−	−	−	−	−	−
6	+	−	−	+	−	−	+	+	−−	−	+	−	−	+	−	−	−	−	+	−	−	−	−	−	−	−	−	+	+	+	−	−	−	−	−	−	−	−	−
7	+	−	+	−	+	−	−	−	−	−	−	−	+	+	−	−	−	−	+	−	−	−	−	−	−	−	−	−	−	−	−	−	−	−	−	−	−	−	−
8	+	−	+	+	−	−	+	+	+	−	−	−	−	−	+	−	−	−	+	−	−	+	−	−	−	−	−−	−	−	−	−	−	−	−	−	−	−	−	−
9	+	−	+	−	−	−	−	−	+	−	−	−	−	−	+	−	−	−	+	−	+	+	−	−	+	+	−	−	−	−	−	−	−	−	−	−	−	−	−
10	+	−	−	+	−	−	−	−	−	+	−	−	−	−	−	−	−	−	−	−	−	−	−	+	−	−	−	−	−	+	−	−	−	−	+	+	−	−	−
11	+	−	+	−	−	−	−	−	+	−	−	−	−	−	+	−	−	−	+	−	−	+	−	−	−	−	−	−	−	−	−	−	−	−	−	−	−	−	−
12	+	−	−	−	−	−	−	−	−	−	−	−	−	−	−	−	−	−	+	−	+	+	−	−	−	−	−	−	−	−	−	−	−	−	−	−	−	−	−
13	+	−	−	−	−	−	−	−	−	−	−	−	−	−	−	−	−	−	+	−	+	−	−	−	−	−	+	−	−	−	−	−	−	−	−	−	−	−	−
14	+	−	−	−	−	−	+	+	−	−	−	−	−	−	+	−	−	−	+	−	−	−	−	−	−	−	−	−	−	−	−	−	−	−	−	−	−	−	−
15	+	−	−	−	−	−	−	−	−	−	+	−	−	−	+	−	−	−	+	−	−	−	−	−	−	−	−	+	+	−	−	−	−	−	−	−	−	−	−
16	+	+	−−	−	−	−	+	+	−	+	+	−	−	−	+	+	+	−	+	−	+	−	−	−	−	−	−	−	−	−	−	−	+	−	−	−	−	−	−
17	+	+	−	+	−	−	+	+	−	+	−	−	−	−	+	+	+	−	+	−	−	−	−	−	−	−	−	−	−	−	−	−	−	−	−	−	−	−	−
18	+	+	−	+	−	−	+	+	−	−	−	−	−	−	+	−	−	−	+	−	−	−	−	−	−	−	−	−	−	−	−	−	−	−	−	−	−	−	−
19	+	+	+	+	−	−	+	+	−	+	−	−	−	+	+	+	+	−	+	−	−	−	−	−	−	−	−	−	−	−	−	−	−	−	−	−	−	−	−
20	+	+	+	−	−	−	+	+	−	−	+	−	−	−	+	+	+	−	+	−	−	−	−	−	−	−	−	−	−	−	−	−	−	−	−	−	−	−	−
21	+	−	+	+	−	−	+	+	−	−	+	−	−	−	+	−	−	−	+	−	−	−	−	−	+	−	−	−	−	−	−	−	−	−	−	−	−	−	−
22	−	−	+	−	−	−	+	+	−	−	−	−	−	−	+	+	+	−	+	−	+	−	−	−	−	−	−	+	+	−	−	−	−	−	−	−	−	−	−
23	+	+	−	−	−	−	−	−	−	−	+	−	−	−	+	+	+	−	−	−	+	−	−	−	−	−	−	+	+	−	−	−	−	−	−	−	−	−	−
24	−	+	+	−	−	−	+	+	−	−	−	−	−	−	+	+	+	−	+	−	+	−	−	−	−	−	−	+	−	+	−	−	−	−	−	−	−	−	−
25	+	−	+	−	−	−	+	+	−	−	−	−	−	+	+	−	−	−	+	−	−	−	−	−	−	−	−	−	−	+	−	−	−	−	−	−	−	−	−
26	−	−	+	−	+	−	+	+	−	−	−	−	−	+	+	−	−	−	−	−	−	−	−	−	−	−	−	−	−	−	−	−	−	+	−	−	−	−	−
27	−	−	−	+	−	−	+	+	−	−	−	−	−	+	−	−	−	−	−	+	−	−	−	−	−	−	−	−	−	+	−	−	−	+	−	−	+	+	−
28	−	−	−	+	−	−	−	−	−	−	−	+	−	+	+	−	−	−	+	−	+	−	+	−	−	−	−	−	−	+	−	−	−	−	−	−	+	+	+
29	−	−	+	+	−	−	+	+	−	−	−	−	−	+	+	−	−	+	−	−	−	−	+	−	−	−	−	−	−	+−	−	−	−	−	−	−	+	+	−
30	−	−	−	+	−	−	−	−	−	−	−	−	−	−	−	−	−	−	+	−	−	−	+	−	−	−	−	−	−	−	−	+	−	−	−	−	+	+	+
31	−	−	−	+	−	−	+	+	−	−	−	−	−	+	+	−	−	+	+	−	+	−	+	−	−	−	−	−	−	+	−	−	−	−	−	−	+	+	+
32	−	−	−	+	−	−	−	−	−	−	−	+	−	+	+	−	++	−	−	−	−	+	−	−	−	−	−	−	+	−	−	−	−	−	−	+	+	+−
33	−	−	−	+	−	−	+	+	−	−	−	+	−	−	−	−	−	+	−	+	−	−	−	−	−	−	−	−	−	−	−	−	−	−	−	−	−	−	−
34	−	−	+	+	−	+	−	−	−	−	−	−	−	+	+	−	−	+	+	−	−	−	−	−	−	−	−	−	−	+	−	+	−	−	−	−	+	+	−
35	−	−	+	+	−	−	+	+	−	−	−	+	−	+	+	−	−	+	−	−	−	−	−	−	−	−	−	−	−	−	−	−	−	−	−	−	−	−	−

Note: Alien fish species are represented in bold columns, and the sites with red numbers are distributed in the downstream part of the Karun River Basin.

## Appendix D

**Table A2.** Substrate size in different sites in the Karun River Basin.

Site Code	Substrate Characteristics
1	Cobbles
2	Cobbles
3	Boulders (small)
4	Boulders (small)
5	Boulders (small)
6	Cobbles
7	Cobbles
8	Cobbles
9	Cobbles
10	Gravel (Fine)
11	Boulders (small)
12	Cobbles
13	Boulders (small)
14	Cobbles
15	Boulders (small)
16	Cobbles
17	Cobbles
18	Cobbles
19	Boulders (small)
20	Boulders (small)
21	Cobbles
22	Cobbles
23	Cobbles
24	Boulders (small)
25	Boulders (small)
26	Cobbles
27	Sand
28	Sand
29	Mud
30	Mud
31	Mud
32	Mud
33	Mud
34	Gravel (Coarse)
35	Sand
